# Dialysis circuit clotting in critically ill patients with COVID-19 infection

**DOI:** 10.1186/s12882-021-02357-3

**Published:** 2021-04-20

**Authors:** Benjamin Zhi En Khoo, Regina Shaoying Lim, Yong Pey See, See Cheng Yeo

**Affiliations:** Department of Renal Medicine, Tan Tock Seng Hospital, 11 Jalan Tan Tock Seng, Singapore, 308433 Singapore

**Keywords:** COVID-19, Dialysis, Circuit life, Circuit clotting, Pro-thrombosis

## Abstract

**Background:**

Coronavirus Disease 2019 (COVID-19) infection has been associated with a hypercoagulable state with increased reports of thrombotic events. Acute kidney injury requiring dialysis is common in critically ill patients and circuit clotting compromises efficacy of treatment. This study aims to analyze the circuit life and circuit clotting during continuous kidney replacement therapy (CKRT) and intermittent hemodialysis in patients with and without COVID-19.

**Methods:**

This is a single-center, retrospective cohort study in critically ill patients undergoing CKRT or intermittent hemodialysis between 1 February 2020 to 22 May 2020. Patients in the intensive care unit (ICU) with COVID-19 infection and contemporary controls who tested negative were included. Co-primary outcomes were functional circuit life for patients on CKRT and all circuit clotting events for patients on CKRT and/or intermittent hemodialysis.

**Results:**

Seventy CKRT circuits and 32 intermittent hemodialysis sessions for 12 COVID-19 cases and 22 CKRT circuits and 18 intermittent hemodialysis sessions for 15 controls were analyzed. CKRT circuit clotting was more common in the COVID-19 group compared to the control group (64% vs 36%, *p* = 0.02), despite higher anticoagulation use in the COVID-19 group (41% vs 14%, p = 0.02). Functional CKRT circuit life was similar in COVID-19 patients and controls (median 11 vs 12 h, *p* = 0.69). On Cox regression analysis, circuit clotting was similar with hazard ratio (HR) 1.90 [95% confidence interval (CI): 0.89–4.04]; however, clotting was increased in COVID-19 patients after adjustment for anticoagulation use (HR: 3.31 [95% CI 1.49–7.33]). In patients with COVID-19, CKRT circuits with anticoagulation had a longer circuit life compared to CKRT circuits without anticoagulation (median 22 versus 7 h respectively, *p* <  0.001). Circuit clotting was similar in both groups undergoing intermittent hemodialysis.

**Conclusion:**

Dialysis clotting amongst COVID-19 patients is increased despite more anticoagulation use and the hazard for clotting is greater especially after adjusting for anticoagulation use. Circuit life was suboptimal in COVID-19 patients on circuits without anticoagulation and therefore routine use of anticoagulation amongst COVID-19 patients should be considered whenever possible.

**Supplementary Information:**

The online version contains supplementary material available at 10.1186/s12882-021-02357-3.

## Introduction

Coronavirus Disease 2019 (COVID-19) presents with a wide spectrum of clinical manifestations, ranging from asymptomatic infection to severe pneumonia and acute respiratory distress syndrome, the latter accounting for admissions to the intensive care unit (ICU). Additionally, several salient extra-pulmonary features of COVID-19 infection in the critically ill patient have been recognized and increasingly, reports of pro-thrombotic tendencies associated with the COVID-19 infection across multiple organ systems have also led to concerns of a hypercoagulable state [1, 2] with a coagulation profile that differs from patients with disseminated intravascular coagulation. This is characterized by raised D-dimer and fibrinogen levels, and normal platelet count [3]. The pathogenesis of this phenomenon is incompletely understood, but is thought to be contributed by multiple factors including significant endothelial inflammation, hyperviscosity and pulmonary hypoxemia leading to vasoconstriction [4]. The increased risk of thrombosis in COVID-19 has manifested clinically with an unusually high incidence of deep vein thrombosis [5], pulmonary embolism [5], acute ischemic stroke [6] and rare reports of acute limb ischemia in patients with COVID-19 infection [7].

The rate of acute kidney injury requiring renal replacement therapy (RRT) in critically ill COVID-19 patients is high, with dialysis required in up to 20 to 35% of patients in the ICU in recent reports [8, 9]. Hemodialysis modalities used in COVID-19 include continuous kidney replacement therapy (CKRT) and intermittent dialysis [9]. Anticoagulation is frequently prescribed during dialysis as contact between blood and the thrombogenic extracorporeal dialysis circuit promotes clotting. Frequent circuit clotting may be particularly problematic amongst COVID-19 patients given the concerns of hypercoagulable state in COVID-19 infections. Multiple anecdotal reports have described an increased incidence of dialysis circuit clotting and decreased dialyzer filter life, with recent reports describing unexpected increased circuit clotting during CKRT [4, 10]. However, there is limited data describing the magnitude of the problem, especially the role of anticoagulation in extending dialysis filter life in COVID-19. We therefore undertook this study to evaluate the impact of COVID-19 infection on dialysis circuit clotting in our local population, in particular comparing circuit life and circuit clotting (with and without anticoagulation) in critically ill patients with COVID-19 infection to those without COVID-19 infection undergoing dialysis in the National Centre of Infectious Diseases (NCID).

## Methods

This was a retrospective, single-center cohort study which included all patients admitted to the ICU at NCID from 1 February 2020 to 22 May 2020 and underwent at least one session of CKRT or intermittent dialysis. In this study, patients with confirmed COVID-19 infection, diagnosed by a positive polymerase chain reaction test for SARS-CoV-2, were compared against a control group consisting of contemporary patients with community acquired pneumonia but tested negative for COVID-19. All dialysis circuits in ICU were analyzed. Co-primary outcomes were functional circuit life for patients on CKRT and all circuit clotting events for patients on CKRT and/or intermittent hemodialysis. Circuit clotting was defined as a failure of the extracorporeal circuit requiring unplanned discontinuation of dialysis and filter replacement, not attributed to access malfunction. Definition was standardized across both groups and adjudicated by the investigators to ensure consistency. Vascular access dysfunction was defined as an inability to maintain the prescribed blood flow requiring intervention for fistulae or catheter replacement. Ethics approval and waiver of informed consent were obtained from the National Healthcare Group Institutional Review Board.

CKRT modalities used included continuous veno-venous hemodiafiltration (CVVHDF), continuous veno-venous hemofiltration (CVVH) and slow continuous ultrafiltration (SCUF), using Prismaflex (Baxter, Deerfield, IL, USA) and AN69 HF 0.9m^2^ dialyzers. Pre-dilution replacement fluid was administered. Blood flow rates of 150-200 ml/min were used. CKRT anticoagulation options were regional citrate anticoagulation using an isotonic citrate replacement fluid administered pre-blood pump with citrate doses ranging from 2.5–3.1 mmol/L, unfractionated heparin infusion ranging from 300 to 800 units/hour, or no anticoagulation. Circuit anticoagulation was largely omitted in patients who were on systemic anticoagulation. Systemic anticoagulation was individualized for each patient by the managing team for specific clinical indications related to thromboembolic risk or events, with no fixed protocol for COVID-19 patients during the study period. Intermittent hemodialysis (IHD) modalities used included sustained low efficiency dialysis, conventional hemodialysis and isolated ultrafiltration. Blood flow rates of 150-250 ml/min were used. Unfractionated heparin bolus between 500 and 1000 units followed by infusion between 250 and 500 units/hour was used for anticoagulation for IHD and this was similar for both groups. Saline flushes were used in patients at high risk of bleeding where heparin was contraindicated. Access used for dialysis included existing arteriovenous fistulae and tunneled dialysis catheters for prevalent ESRD patients. In patients without existing access, non-tunneled dialysis catheters 12 Fr – 12.5 Fr in diameter with lengths of 16-20 cm depending on insertion site were used.

Baseline demographics, significant comorbidities and clinical data for each patient were obtained from the electronic health records. In addition, dialysis-related parameters for each CKRT or intermittent dialysis circuit were obtained from the dialysis flow chart. Filtration fraction was defined as ultrafiltration flow rate divided by plasma flow rate in line with previous definitions, wherein ultrafiltration flow rate was the sum of the total replacement flow rate and the net ultrafiltration and plasma flow rate was equal to blood flow rate x (1 – Hematocrit) + replacement flow rate (pre-dilution) [11].

Results were reported as frequency (percentages), mean ± standard deviation or median with interquartile range, as appropriate. Differences between groups were analyzed using chi-square test or Fisher’s exact test for categorical variables and independent student t-test or Mann-Whitney U test was performed for continuous variables, as appropriate. Cox proportional hazard regression model was used to examine the primary outcome of circuit life and proportional hazard assumption for Cox regression was tested both graphically and using Schoenfeld residuals.

The Kaplan-Meier survival curves for dialyzer circuit life were plotted and analyzed using the log-rank test. CKRT circuits which did not clot but terminated for other reasons were censored. A *p*-value of < 0.05 was considered statistically significant and all analyses were performed using the software Stata (version 14.0, StataCorp LP, College Station, TX, USA).

## Results

A total of 27 subjects (12 COVID-19 patients and 15 control patients) underwent dialysis during the study period. The baseline characteristics of the patients are summarized in Table 1. More patients with end-stage kidney disease (ESKD) were in the control group compared to the COVID-19 group but this was not statistically significant (53.3% vs 16.7%, *p* = 0.10).

**Table 1:** Baseline characteristics of COVID-19 patients and controls

	COVID-19	Control	*p*-value
Number of CKRT circuits	70	22	Not applicable
Number of patients	12	15	
CKRT circuits per patient	10 (2, 10)	2 (2, 3.5)	
Duration of CKRT, days	6.5 (3, 17.5)	3 (2, 3.5)	
Number of circuits per patient-day of CKRT	0.79 (0.49, 1.15)	1 (0.78, 1)	
Number of intermittent dialysis sessions	32	18	
Age, years	65 ± 14	58 ± 9	0.16
Male gender, n (%)	9 (75)	13 (87)	0.39
ESKD, n (%)	2 (17)	8 (53)	0.10
Weight, kg	69 ± 17	73 ± 18	0.56
SOFA^a^	10 ± 5	8 ± 4	0.23
Comorbidities			
HTN, n (%)	8 (67)	10 (67)	1.00
IHD, n (%)	4 (33)	10 (67)	0.13
DM, n (%)	2 (17)	11 (73)	< 0.05
Baseline medication use			
Antiplatelet, n (%)	3 (25)	8 (53)	0.43
Anticoagulant^b^, n (%)	1 (8)	1 (7)	

*Abbreviations*: *CKRT* Continuous kidney replacement therapy, *ESKD* End stage kidney disease, *SOFA* Sequential organ failure assessment, *HTN* Hypertension, *IHD* Ischemic heart disease, *DM* Diabetes mellitus

^a^ SOFA score, is taken at time of dialysis initiation

^b^ Any vitamin K antagonists, direct acting oral anticoagulants or low molecular weight heparin

In total, 70 CKRT circuits in the COVID-19 group and 22 CKRT circuits in the control group were utilized. The characteristics of the CKRT circuits in COVID-19 and control patients are shown separately in Table 2. 56% of the CKRT circuits in the COVID-19 group used an internal jugular vein as the dialysis vascular access compared with only 9% in the control group, with the remaining circuits using a femoral vein as dialysis vascular access. CVVHDF was the most common CKRT modality accounting for more than 70% of circuits used in both groups. Overall, the use of anticoagulation (either circuit or systemic) was more common in the COVID-19 group compared to controls (41% vs 14% respectively, *p* = 0.02). Regional citrate anticoagulation was the most common anticoagulation used in COVID-19 patients accounting for 21 circuits (30%).

**Table 2:** Characteristics of CKRT circuits for COVID-19 patients and controls

	COVID-19 (*n* = 70)	Control (*n* = 22)	*p*-value
Premature termination of dialysis due to clotting, n (%)	45 (64)	8 (36)	0.02
Circuit life, hours	11 (5,18)	12 (7,24)	0.69
CKRT modality, n (%)			
CVVHDF	56 (80)	16 (73)	0.53
CVVH	13 (19)	6 (27)	
SCUF	1 (1)	0 (0)	
Systemic anticoagulation, n (%)	8 (11)	0 (0)	1.00
Anticoagulation during dialysis, n (%)	24 (34)	3 (14)	0.10
- Regional citrate anticoagulation	21 (30)	3 (14)	
- Heparin	3 (4)	0 (0)	
Systemic anticoagulation or anticoagulation during dialysis, n (%)	29 (41)	3 (14)	0.02
Prone positioning, n (%)	12 (17)	0 (0)	0.03
Vascular access, n (%)			
- Femoral non-tunneled	31 (44)	20 (91)	< 0.05
- Internal jugular non-tunneled	39 (56)	0 (0)	
- Internal jugular tunneled	0 (0)	2 (9)	
Blood flow rate, ml/min	155.0 ± 13.6	151.4 ± 6.4	0.22
Effluent dose, ml/kg/hr	33.7 ± 6.9	27.3 ± 9.4	< 0.05
Filtration fraction, %	14.8 ± 5.2	18.4 ± 5.2	0.01
Hematocrit, %	24 ± 3.9	34.8 ± 7.6	< 0.05
Platelets, × 10^9^/L	136 ± 76	134 ± 78	0.90
INR	1.2 (1, 1.4)	1.3 (0.7, 1.9)	< 0.05
aPTT, seconds	36.0 (29.6, 42.4)	35.1 (22.6, 47.6)	0.84

*CKRT* Continuous Kidney Replacement Therapy, *CVVHDF* Continuous veno-venous hemodiafiltration, *CVVH* Continuous veno-venous hemofiltration, *SCUF* Slow continuous ultrafiltration, *INR* International normalized ratio, *aPTT* Activated partial thromboplastin time

**Fig. 1:**
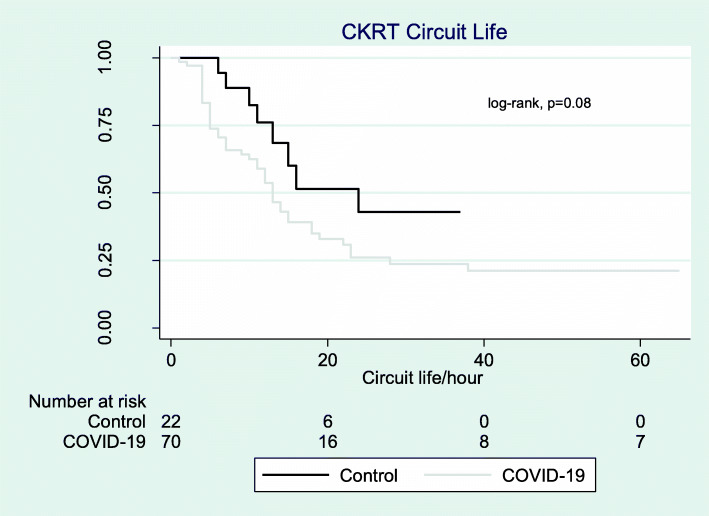
CKRT Circuit Life in COVID-19 Patients and Controls. CKRT: Continuous Kidney Replacement Therapy. Kaplan-Meier survival curve of all continuous kidney replacement therapy circuits comparing between COVID-19 patients and control. There is a trend towards lower circuit survival amongst COVID-19 patients compared to control but this is not statistically significant (*p* = 0.08)

Circuit clotting was more frequent in COVID-19 group, with 45 out of 70 circuits clotted in COVID-19 group compared to 8 out of 22 circuits in the control group (64% vs 36% respectively, *p* = 0.02) despite more anticoagulation use in COVID-19 group (41% vs 14% respectively, *p* = 0.02). The median circuit life was however similar between COVID-19 group and control group (circuit life: 11 vs 12 h, *p* = 0.69). The Kaplan-Meier survival curve for dialyzer circuit life showed separation between the COVID-19 patients and control group but this did not reach statistical significance, with log rank test of *p* = 0.08 (Fig. 1). On Cox regression analysis, the unadjusted hazard ratio (HR) of circuit clotting in COVID-19 patients compared to controls was (HR 1.9 [95% Confidence Interval C.I.: 0.89–4.04]).

**Fig. 2:**
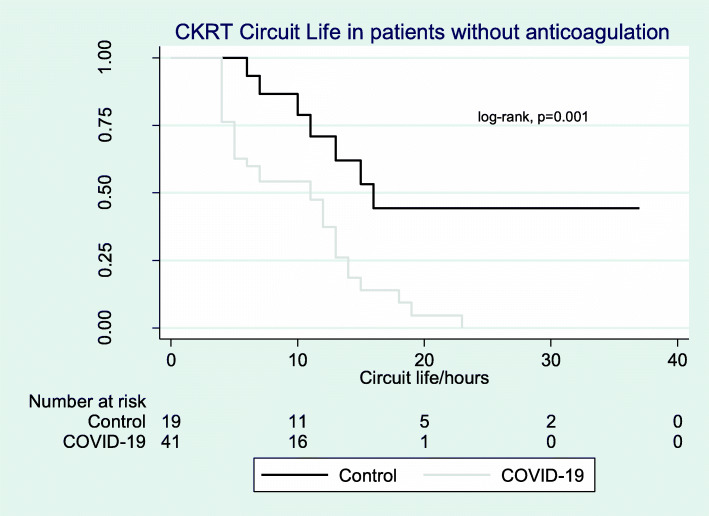
CKRT Circuit Life without Anticoagulation in Patients with COVID-19 and Controls. CKRT: Continuous Kidney Replacement Therapy. Kaplan-Meier survival curve of continuous kidney replacement therapy circuit comparing between COVID-19 patients and control in a subgroup which did not receive systemic or regional anticoagulation. In the absence of anticoagulation, there is significantly reduced survival of circuit amongst COVID-19 patients compared to control (*p* = 0.001)

However, after adjusting for use of anticoagulation, the hazards for CKRT circuit clotting was higher in COVID-19 patients (HR 3.31, [95% C.I.: 1.49–7.33]) compared to controls. Comparing CKRT circuits without anticoagulation, clotting was more common in COVID 19 patients compared to controls (76% vs 37%, *p* <  0.005). Comparing all CKRT circuits with no anticoagulation, circuit survival was significantly shorter in patients with COVID-19 compared to controls (log-rank *p* = 0.001) (Fig. 2). The use of anticoagulation was associated with significantly increased median circuit life amongst COVID-19 patients (22 [Interquartile Range 9–40] versus 7 [Interquartile Range 4–12] hours respectively, *p* < 0.001). This was generally well tolerated with total calcium/ionized calcium ratio not exceeding 2.5 for all patients.

A total of 32 sessions of IHD were performed in the COVID-19 group and 18 sessions were performed in the control group. Anti-coagulation use was similar in both groups (34% vs 22% respectively, *p* = 0.52). Hemodialysis circuit characteristics are presented in Table 3. There was no difference between circuit clotting in the patients on intermittent dialysis with COVID-19 compared to control group (38% vs 33% respectively, *p* = 0.77).

**Table 3:** Characteristics of intermittent dialysis circuits for COVID-19 patients and controls

	COVID-19 (*n* = 32)	Control (*n* = 18)	*p*-value
Number of dialysis circuits clotted, n (%)	12 (38)	6 (33)	0.77
Modality, n (%)			
- SLED	13 (41)	10 (56)	0.57
- IHD	16 (50)	6 (33)	
- Others^a^	3 (9)	2 (11)	
Systemic anticoagulation, n (%)	6 (19)	0 (0)	0.08
Systemic anticoagulation and/or anticoagulation (heparin) during dialysis, n (%)	11 (34)	4 (22)	0.52
Vascular access, n (%)			
- Non-tunneled catheter	19 (59)	7 (39)	0.01
- Tunneled catheter	0 (0)	5 (28)	
- Arteriovenous fistula	13 (41)	6 (33)	
Blood flow rate, ml/min	206 ± 49	174 ± 42	0.02
Dialysate flow rate, ml/min	372 ± 155	322 ± 148	0.27
Hematocrit, %	25.8 ± 3.7	27.9 ± 5.3	0.11
Platelet, ×10^9^/L	406 ± 105	207 ± 89	< 0.001
INR	1.1 (1, 1.2)	1.2 (1, 1.4)	0.53
aPTT, seconds	37.9 (18.6, 57.2)	36.6 (24.8, 48.4)	0.12

*Abbreviations*: *SLED* Sustained low efficiency dialysis, *IHD* Intermittent hemodialysis, *INR* International normalized ratio, *aPTT* Activated partial thromboplastin time

^a^ Others include isolated ultrafiltration

With regards to vascular access, there was no access dysfunction in patients with established arteriovenous fistulae. Dialysis catheters were dysfunctional in a small minority of patients in both groups, and this was treated by catheter replacement. In our study, seven patients (58.3%) critically ill with COVID-19 who required dialysis died. Of the three survivors who had acute kidney injury requiring dialysis, renal replacement therapy was not required at the time of discharge.

## Discussion

This report highlights the overall high frequency of dialyzer circuit clotting in COVID-19 patients, occurring in up to 64% of CKRT circuits. This is consistent with recently published reports where between 40 and 97% of CKRT circuits in patients with COVID-19 infection experienced unexpected or premature circuit clotting [4, 10]. CKRT circuit clotting was particularly more common amongst COVID-19 patients compared to controls, despite more frequent use of anticoagulation amongst the COVID-19 patients and higher international normalized ratio in the control group. The latter is explained by two patients in the control group with severe disseminated intravascular coagulation. While the median circuit life between the two groups was similar, we recognize the sample was small and may not be powered to detect any difference. Additionally, in COVID-19 patients, CKRT circuits without anticoagulation had a significantly shorter circuit life compared to circuits with anticoagulation, consistent with previously published data [10]. Circuit anticoagulation should thus be strongly considered for patients with COVID-19 with the aim of prolonging circuit life. Overall, CKRT circuit clotting is associated with reduction in the delivered dialysis dose and thus the efficacy of treatment [12]. Beyond that, dialyzer circuit clotting leads to increased nursing encounters to troubleshoot and/or re-prime circuits, which is less desirous from an infection control viewpoint during this pandemic.

Intermittent HD including conventional hemodialysis and sustained low efficiency dialysis may be considered in patients with COVID-19 infection who are hemodynamically stable. Intermittent HD is performed over a shorter period with higher blood and dialysate flows and has been shown in the general population to have a lower incidence of circuit clotting compared to CKRT [13]. We used low dose heparin or regular saline flushes [14] if heparin was contraindicated to minimize circuit clotting. While there was no significant difference in clotting in patients with COVID-19 and the control group, the numbers were small and it is not clear if a larger number in a prospective study may show a difference.

The strengths of this study are the inclusion of a contemporary control group to allow for direct comparison of the impact of COVID-19 on dialyzer circuit clotting. The COVID-19 patients and control group were recruited from the same ICU over the same period therefore reducing the potential variability in dialysis nursing care, dialysis prescription and anticoagulation use protocol. There are however limitations to this study including the small sample size. Although we analyzed all consecutive patients admitted to the ICU over an extended period, there were only 27 subjects in our study. Hence, we undertook to analyze each dialyzer circuit use to better understand the magnitude of the problem and the effect of anticoagulation. This study is also retrospective and observational in nature and therefore biases and unmeasured confounders between the COVID-19 and control group may not be accounted for. As illness acuity and other extraneous factors may affect circuit life and circuit clotting, adjusting for these in the COVID-19 group and control group may be useful for future studies, in which controls are better selected through propensity-score matching or further covariates in a multivariable model. The overall circuit life for CKRT circuits documented is also generally short which could reflect the lower use of anticoagulation in the initial phase of the pandemic. It is not clear if delayed alarm response due to the need to don personal protective equipment and other measures may also contribute to reduced dialyzer circuit life. Lastly, this is a single-center study and therefore limits the generalizability of the study.

## Conclusion

Dialysis circuit clotting is common amongst COVID-19 patients. While circuit life is similar in patients undergoing CKRT with COVID-19 infection compared to a control group, overall incidence of circuit clotting is increased. The risk of circuit clotting is greater amongst COVID-19 patients not receiving anticoagulation compared to control. Therefore, it is necessary to ensure appropriate anticoagulation whenever possible in COVID-19 patients.

## Supplementary Information

**Additional file 1**. Regional Citrate Anticoagulation Protocol Used for Continuous Kidney Replacement Therapy.

## Data Availability

The datasets used and/or analyzed during the current study are available from the corresponding author on reasonable request.

## Abbreviations

CI: Confidence interval
CKRT: Continuous kidney replacement therapy
COVID-19: Coronavirus Disease 2019
CVVH: Continuous veno-venous hemofiltration
CVVHDF: Continuous veno-venous hemodiafiltration
ESKD: End-stage kidney disease
HR: Hazard ratio
IHD: Intermittent hemodialysis
ICU: Intensive care unit
RRT: Renal Replacement Therapy
SCUF: Slow continuous ultrafiltration
NCID: National Centre of Infectious Diseases
