# Supplementary material for: Dialysis circuit clotting in critically ill patients with COVID-19 infection
Source: BMC Nephrol. 2021 Apr 20;22:141. doi: 10.1186/s12882-021-02357-3 (PMC8056790; doi:10.1186/s12882-021-02357-3)
Supplement: Supplementary file 1 — Additional file 1. Regional Citrate Anticoagulation Protocol Used for Continuous Kidney Replacement Therapy. [file 12882_2021_2357_MOESM1_ESM.docx]

Regional Citrate Anticoagulation Protocol Used for Continuous Renal Replacement Therapy

Calcium Gluconate Infusion

Blood pump

Pre-dilution Citrate Replacement

18mmol/L

Effluent

Dialyzer

From patient

To patient

Calcium-free Dialysate

Supplementary Figure 1: Schematic of Circuit for Continuous Renal Replacement Therapy using Regional Citrate Anticoagulation

| Fluid Used | Sodium (mmol/L) | Chloride (mmol/L) | Citrate (mmol/L) | Potassium (mmol/L) | Magnesium (mmol/L) | Phosphate  (mmol/L) | Bicarbonate  (mmol/L) | Calcium (mmol/L) |
| --- | --- | --- | --- | --- | --- | --- | --- | --- |
| Pre-dilution Replacement:  Regiocit (Isotonic Citrate) | 140 | 86 | 18 | Added as sliding scale based on serum value | 0 | 0 | 0 | 0 |
| Dialysate:  Biphozyl (Calcium-free) | 140 | 122 | 0 | 4 | 0.75 | 1 | 22 | 0 |

Supplementary Table 1: Fluid used for Regional Citrate Anticoagulation and Electrolyte Composition

Initial blood flow rates: 150ml/min

Initial citrate dose: 2.5mmol/L

Initial dose of replacement fluid = Desired Citrate Dose / Fluid Citrate Concentration x Blood Flow (in ml/min)

- With blood flow at 150ml/min, dose of isotonic citrate replacement would be 2.5 / 18 x 150 x 60 = 1250ml/min

Target circuit (post-filter) calcium: Below 0.4mmol/L

- If circuit calcium is persistently high without evidence of citrate accumulation, citrate dose will be increased

Calcium replacement: Calcium gluconate infusion, titrated to keep serum ionized calcium 1-1.2mmol/L

Machine used: Prismaflex
